# Supplementary material for: Exogenous Ceramide Serves as a Precursor to Endogenous Ceramide Synthesis and as a Modulator of Keratinocyte Differentiation
Source: Cells. 2022 May 25;11(11):1742. doi: 10.3390/cells11111742 (PMC9179460; doi:10.3390/cells11111742)
Supplement: Supplementary file 1 [file cells-11-01742-s001.zip › cells-1712894-supplementary.pdf]

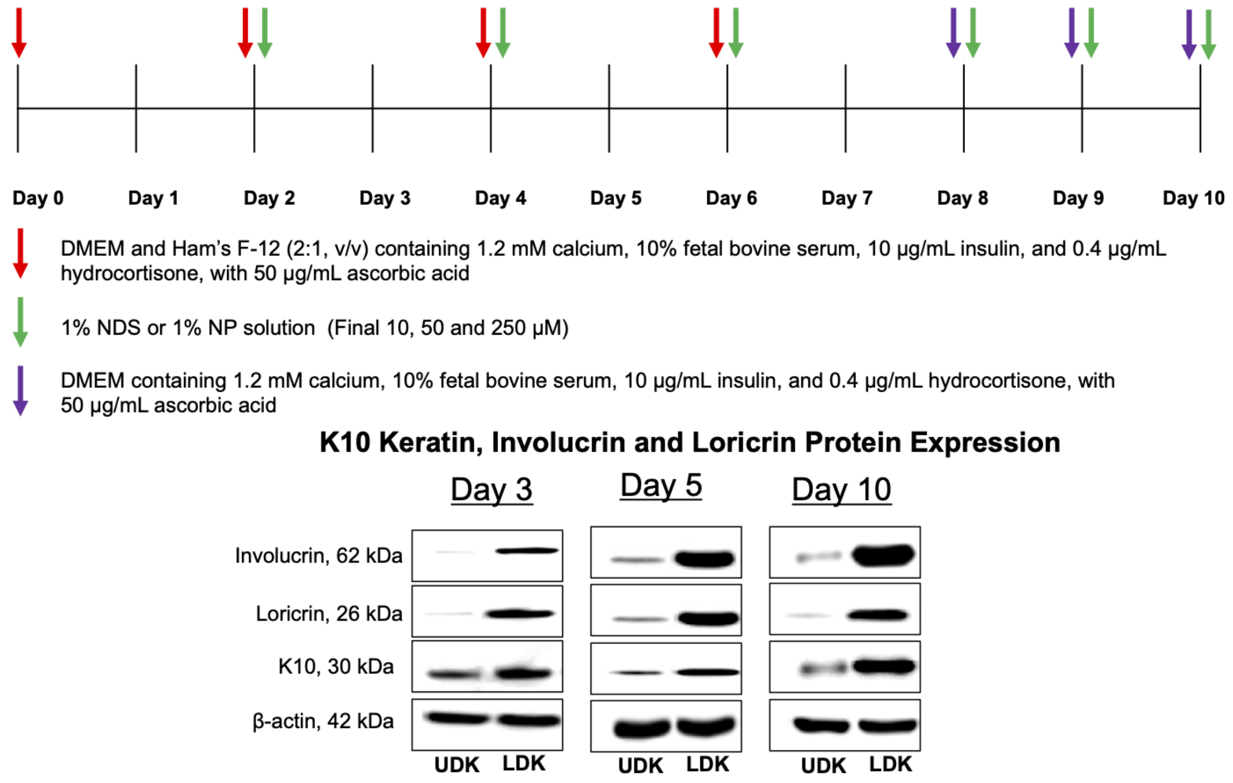

Figure S1: Culture Scheme.
KCs cultured with serum-free cultured medium containing 0.07 mM Ca^{2+} at 60-70% confluence were further cultured in differentiation-inducing medium as indicated scheme. Western blot analysis demonstrated that increases in K10 keratin (early KC differentiation marker), involucrin (middle KC differentiation marker) and loricrin (late KC differentiation marker) production during cultures.

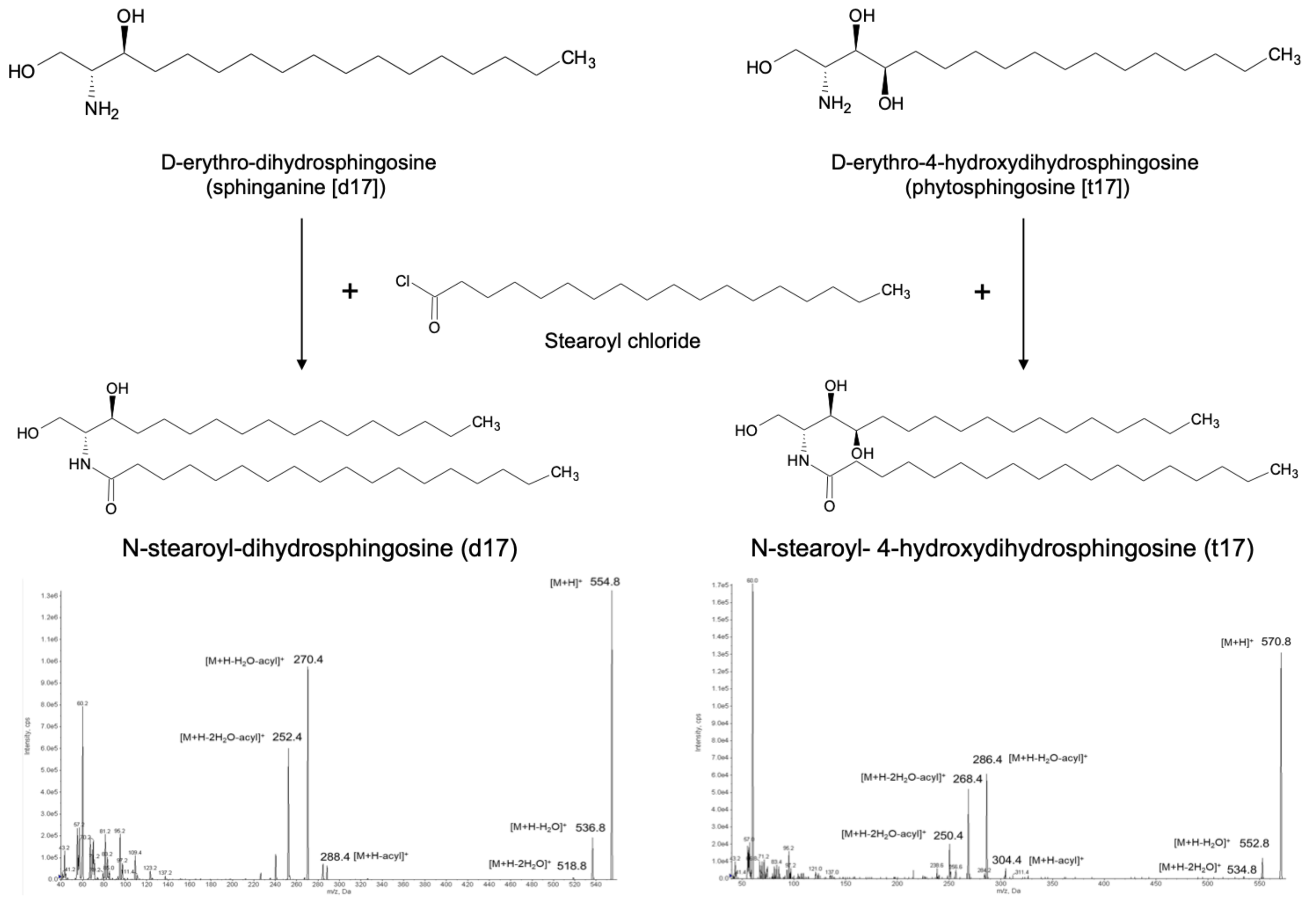

Figure S2: Outline of C17NDS and C17 NP preparation and verification by LC-MS/MS.

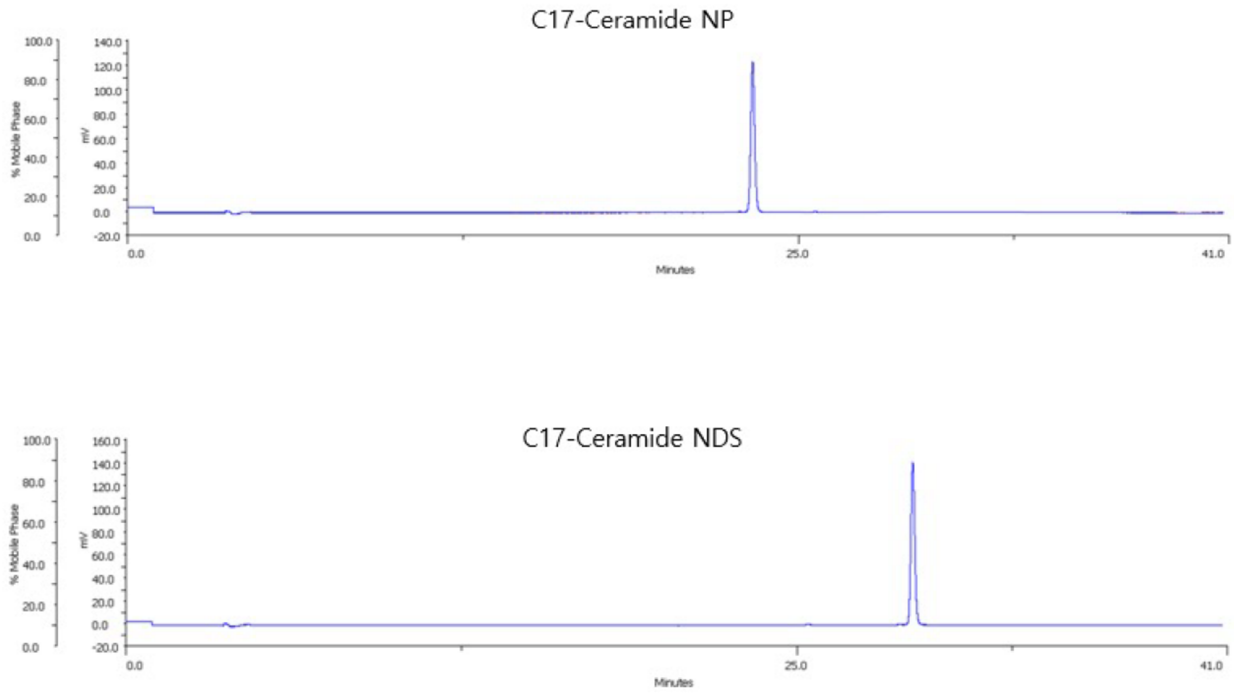

Figure S3: HPLC choromatograms syntheisized C17NDS.

	Precursor of diverse Cer	Differentiation	Diverse Cer via differentiation	Cathelicidin
**NDS**	○	◎	○	○
**NP**	X	○	○	X

	Invoruclin	Loricrin
**NDS**	◎	◎
**NP**	○	○

Figure S4: Biological features of exogenous NDS and NP in KC.
